# Supplementary material for: The genome sequence of the Drinker, Euthrix potatoria (Linnaeus, 1758) (Lepidoptera: Lasiocampidae)
Source: Wellcome Open Res. 2025 Oct 3;10:534. [Version 1] doi: 10.12688/wellcomeopenres.24922.1 (PMC12770893; doi:10.12688/wellcomeopenres.24922.1)
Supplement: Peer review history

# Reviewer report: Reviewer response for version 1

**DOI:** 10.21956/wellcomeopenres.27452.r142944

**Published:** 2026-01-27

**Authors:** Aleksandra Gwiazdowska

**Affiliations:** 1 Polish Academy of Sciences, Twarda, Poland

The authors described the genome of the Drinker ( *Euthrix potatoria*). The introduction briefly described the species. The photograph of the Drinker is good quality, but the moth is not visible in this horizontal position. The methods are described in great detail, and each part has a link to see the details on the web side, so the most important information is easy to find. The pipeline and the accession number are  also available online. 

 In summary, the results and methods are available and reproducible. The reference genome will help for future studies.

Are sufficient details of methods and materials provided to allow replication by others?

Yes

Is the rationale for creating the dataset(s) clearly described?

Yes

Are the datasets clearly presented in a useable and accessible format?

Yes

Are the protocols appropriate and is the work technically sound?

Yes

Reviewer Expertise:

Population Genetics, Conservation Genetics

I confirm that I have read this submission and believe that I have an appropriate level of expertise to confirm that it is of an acceptable scientific standard.

# Reviewer report: Reviewer response for version 1

**DOI:** 10.21956/wellcomeopenres.27452.r142945

**Published:** 2026-01-05

**Authors:** Aqeel Alyousuf

**Affiliations:** 1 University of Basrah, Basrah, Iraq

The manuscript reports a high-quality, chromosome-scale genome assembly generated using PacBio HiFi sequencing and Hi-C scaffolding. The study is well organized, and the laboratory procedures and bioinformatic analyses are described clearly and rigorously. Assembly quality is supported by comprehensive validation metrics, and the inclusion of the mitochondrial genome further strengthens the completeness of the assembly. A minor recommendation is to introduce the full species name at first mention and subsequently use the abbreviated form ( *E. potatoria*), in accordance with standard taxonomic practice.

Are sufficient details of methods and materials provided to allow replication by others?

Yes

Is the rationale for creating the dataset(s) clearly described?

Yes

Are the datasets clearly presented in a useable and accessible format?

Yes

Are the protocols appropriate and is the work technically sound?

Yes

Reviewer Expertise:

Entomology

I confirm that I have read this submission and believe that I have an appropriate level of expertise to confirm that it is of an acceptable scientific standard.

# Reviewer report: Reviewer response for version 1

**DOI:** 10.21956/wellcomeopenres.27452.r138884

**Published:** 2025-12-08

**Authors:** Masaki Takenaka

**Affiliations:** 1 University of Tsukuba, Tsukuba, Ibaraki, Japan

There were no major issues. The data can be considered sufficiently valuable.

 Only three minor points are noted:

- What is the concentration of ethanol?
- How was the DNA extracted for analysis of mtDNA COI region?
- COI refers to mitochondrial DNA. Please describe it accurately as “mtDNA COI region,” and spell it out in full at first mention.

Are sufficient details of methods and materials provided to allow replication by others?

Yes

Is the rationale for creating the dataset(s) clearly described?

Yes

Are the datasets clearly presented in a useable and accessible format?

Yes

Are the protocols appropriate and is the work technically sound?

Yes

Reviewer Expertise:

Entomology

I confirm that I have read this submission and believe that I have an appropriate level of expertise to confirm that it is of an acceptable scientific standard.
